# Albumin Binds Doxorubicin via Self–Assembling Dyes as Specific Polymolecular Ligands

**DOI:** 10.3390/ijms23095033

**Published:** 2022-05-01

**Authors:** Anna Jagusiak, Katarzyna Chłopaś, Grzegorz Zemanek, Izabela Kościk, Paweł Skorek, Barbara Stopa

**Affiliations:** 1 Chair of Medical Biochemistry, Faculty of Medicine, Medical College, Jagiellonian University, 31-034 Krakow, Poland; grzegorz.zemanek@uj.edu.pl (G.Z.); izabela.koscik@uj.edu.pl (I.K.); barbara.stopa@uj.edu.pl (B.S.); 2 Pulmonology and Allergology Clinical Department, University Hospital in Krakow, 30-688 Krakow, Poland; kchlopas@su.krakow.pl; 3 Faculty of Biotechnology and Horticulture, University of Agriculture in Krakow, 31-425 Krakow, Poland; 4 Department of Cardiac and Vascular Diseases, John Paul II Hospital, 31-202 Krakow, Poland; pawel.skorek23@gmail.com

**Keywords:** drug delivery systems (DDS), self–assembled ribbon–like structures (SRLS), Congo red (CR), doxorubicin (Dox), bovine serum albumin (BSA), human serum albumin (HSA), dynamic light scattering (DLS), elution volume (V_e_)

## Abstract

Congo red (CR) type self–assembled ribbon–like structures (SRLS) were previously shown to interact with some proteins, including albumin. SRLS also complex with some drugs with a flat, ring–shaped structure with aromatic characteristics, intercalating them into their ribbon structure. The combination of interaction with proteins and drug binding by SRLS enables the use of such systems for immunotargeting. It is especially interesting in the case of chemotherapeutic agents. The present experiments aimed to show that the model carrier system composed of supramolecular albumin and Congo red efficiently binds doxorubicin (Dox) and that the drug can be released at reduced pH. The presented results come from the studies on such complexes differing in the molar ratio of CR to Dox. The following methods were used for the analysis: electrophoresis, dialysis, gel filtration, spectral analysis, and analysis of the size of the hydrodynamic radius using the dynamic light scattering method (DLS). The applied methods confirmed the formation of the CR–Dox complex, with large dimensions and changed properties compared with free CR. The presented results show that albumin binds both CR and its complex with Dox. Various CR–Dox molar ratios, 5:1, 2:1, and 1:1, were analyzed. The confirmation of the possibility of releasing the drug from the carriers thus formed was also obtained. The presented research is important due to the search for optimal solutions for the use of SRLS in drug immunotargeting, with particular emphasis on chemotherapeutic agents.

## 1. Introduction

Carriers, in particular drug delivery systems (DDS), by binding the drug, reduce its toxicity, enable targeted transport, and controlled dosing [1,2]. Among the numerous nanocarriers for drugs, nanosystems incorporating albumin as a building element are a widely studied group. These systems include albumin nanoparticles, nanospheres, albumin–coated liposomes, microbubbles, nanocapsules, and many others [3]. These systems can be of various sizes and shapes. Their advantage consists of the possibility of introducing modifications by adding ligands that facilitate the delivery of the carrier to the appropriate molecular target. Additionally, albumin is a protein capable of binding a wide variety of compounds [4,5], including Congo red (CR) ribbon supramolecular systems (SRLS) capable of complexing various drugs. Currently, drugs are sought for the therapy of molecular targets. The combination of targeted immunotherapy with the additional possibility of targeted drug delivery is also interesting [6,7]. Many drugs are poorly soluble substances, unstable in the body’s environment, with a short half–life. Others are water–soluble compounds, but the doses used have to be limited due to strong cytotoxic activity. The chemotherapeutic agents used in the treatment of neoplasms are characterized by low selectivity. The side effects affecting healthy tissues make the search for the solutions an urgent need to reduce the toxicity of drugs, not only by lowering the dose but primarily through targeted action technologies while maintaining the therapeutic dose [8,9], especially because the effectiveness of pharmacotherapy depends strongly on the local concentration of the drug and the length of contact time with the target site. It is important to develop safe solutions that will increase the effectiveness of targeted therapies, as well as to develop efficient and safe carriers of anti–inflammatory and anti–cancer drugs [10].

Albumin particles accumulate preferentially in tumors and in inflammatory sites, which is an important advantage for their use as a drug carrier [11]. This is explained by the increased metabolism of cancer cells and their increased demand for proteins. Albumin nanoparticles accumulate in tumor tissue both through passive and active targeting, and therefore, albumin nanoparticles have a high therapeutic potential. Most tumors are supplied with blood, and their venous network is chaotically branched and much more permeable. Moreover, tumors do not have lymphatic drainage that would normally drain albumin that has left the vascular bed. Inflamed and neoplastic tissues are rich in weakened and leaky blood vessels as a result of rapid and chaotic angiogenesis. In such capillaries, the phenomenon of increased permeability and retention (EPR) takes place, which is not observed in the vessels of healthy tissues. The diameter of the gaps between the endothelial cells in the tumor capillaries is 100–1000 nm, while the diameter of the gaps in the normal epithelium is 2–6 nm. This contributes to the increased uptake of nanoparticles by the neoplastic tissue [12]. The described gap sizes also allow for the increased penetration of macromolecules by passive transport into the neoplastic tissue. Due to this phenomenon, the drug–linked albumin easily penetrates the neoplastic tissue, bypassing healthy tissues. An additional factor facilitating the accumulation of albumin in neoplastic cells is the high expression of the SPARC glycoprotein, or osteonectin—a secreted protein, acidic and rich in cysteine. It is a protein with high homology to albondine (the gp60 receptor for albumin found on endothelial cells) [13].

Albumins, both human serum albumin (HSA) and animal, e.g., bovine serum albumin (BSA), are promising, well–described proteins used as a building component for nanocarriers [14,15,16]. Albumin is a protein with a relatively low molecular weight (66 kDa) and high concentration in the blood. Albumin is highly bioavailable, biocompatible, non–toxic, and exhibits low immunogenicity [3]. This protein is rich in cysteine [17], which provides stability enabling it to endure pH ranging from 4 to 9 [18]. Because of these physicochemical properties, HSA has been first studied as an endogenous binding protein for its marked effects on the pharmacodynamics and pharmacokinetics of small molecular therapeutics [19,20,21,22] and was brought into the spotlight as a drug delivery vehicle. Albumin has many functions. It is responsible for the oncotic pressure of plasma and the transport of carbon dioxide. In addition, it has a pH buffering effect [23]. Albumin is a protein known to bind various compounds (free fatty acids, bilirubin, steroid hormones, calcium and copper ions, as well as drugs: sulfonamides, aspirin, penicillin). Compounds that are easily bound by albumin include numerous natural and synthetic dyes, especially those with hydrophobic and negatively charged properties, e.g., curcumin [24,25].

Albumin works well as a carrier, protecting healthy tissues against the toxic effects of the drugs it carries. It extends the half–life of the drug in the body. At the same time, it easily accumulates in the neoplastic tissue via the phenomenon of passive transport. In addition, albumin can be easily subjected to surface modifications (adding polypeptides, antibodies, or their fragments), which will cause the drug to be delivered by active transport (receptor–dependent endocytosis) [26].

**Figure 1:**
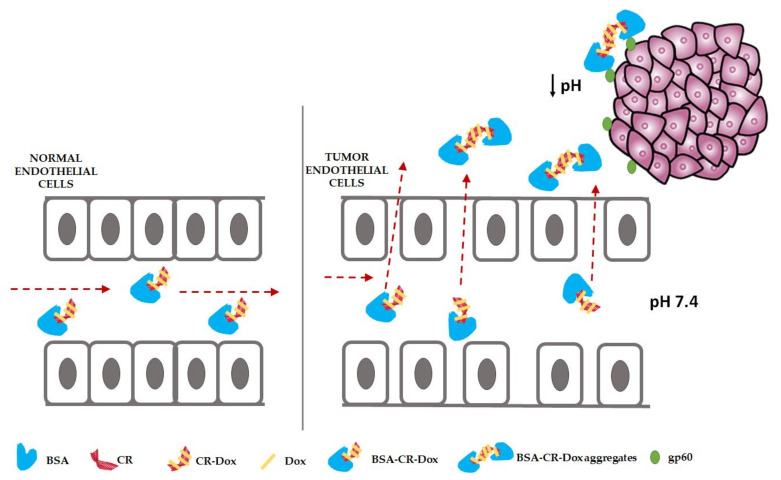
Endothelial cells of a healthy vessel are tightly adjacent to each other vs. capillary in tumor tissue with “relaxed epithelium” (EPR) (albumin–Congo red–doxorubicin: BSA–CR–Dox passive transport). Active transport is mediated by the BSA–CR–Dox binding to SPARC glycoprotein (gp60), which penetrates tumor cells. Additionally, the formation of aggregates after lowering the pH in the tumor environment is visible, which facilitates the release of Dox. Arrows indicate the direction in which the complex is moving.

However, the methodologies of doxorubicin (Dox) binding to albumin described in the literature involve time–consuming and expensive procedures. Hence, the aim of the study was to create a highly desirable, easy–to–synthesize, cheap, and safe carrier system, which will simultaneously ensure effective drug delivery to the neoplastic tissue and enable its release at the target site. Ribbon–like supramolecular systems (SRLS), of which the representative is Congo red, are an example of such systems. Congo red type self–assembled ribbon–like structures (SRLS) were previously shown to interact with some proteins, including albumin [27,28,29]. CR also binds Dox by intercalation. The present study aimed to create a carrier system based on albumin, which by binding the CR–Dox complex transports it to the place where the vessels in the tumor are more permeable, and lowering the pH causes aggregation of the carrier and release of the drug. Passive transport can be assisted by active transport through binding to gp60 (Figure 1).

Because this is a novel study, its potential application in many different areas is thus possible, e.g., in surface modification of biodegradable metal devices or biomedical hydrogels [30,31].

## 2. Results

### 2.1. Dox Forms Complexes with CR (CR–Dox), Which Are Bound by Albumin. Agarose Gel Electrophoresis and Chromatographic Analysis

The analysis aimed to compare the ability of albumin to bind free Dox and CR–Dox complexes prepared in various molar ratios (1:1, 2:1, and 5:1). The CR in sample number 3 forms a complex with BSA, which migrates faster than the free protein. All CR is bound to the protein (10–fold excess of CR over BSA was used). Comparing the migration rate of free CR (sample 1) with the CR: Dox 5:1, 2:1, and 1:1 complexes (samples No 5, 8, and 11), a faster migration of the CR–Dox complexes was observed compared to the free CR. Strong, stable complexes are formed, which in all three cases completely bind the added amounts of Dox. Ingredients in a 2:1 ratio seem to be more complex with each other than in a 5:1 ratio.

**Figure 2:**
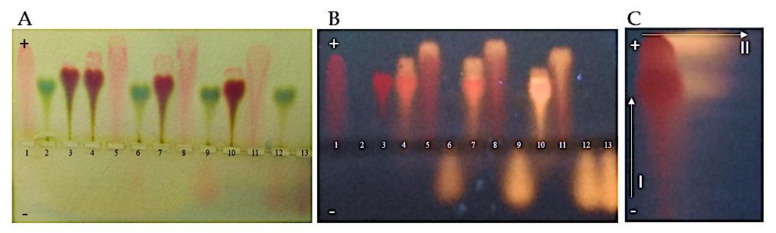
Complexes between BSA and CR–Dox. BSA free and in the complexes with CR or CR–Dox seen as migrating toward the anode; Dox free seen as migrating toward the cathode: (1) CR, (2) BSA, (3) BSA–CR complex, (4) BSA–CR–Dox (CR: Dox molar ratio = 5:1; CR:BSA molar ratio = 10:1), (5) CR–Dox (CR: Dox molar ratio = 5:1), (6) BSA–Dox (Dox concentration as in probes 4 and 5), (7) BSA–CR–Dox (CR: Dox molar ratio = 2:1; CR:BSA molar ratio = 10:1), (8) CR–Dox (CR: Dox molar ratio = 2:1), (9) BSA–Dox (Dox concentration as in probes 7 and 8), (10) BSA–CR–Dox (CR: Dox molar ratio = 1:1; CR:BSA molar ratio = 10:1), (11) CR–Dox (CR: Dox molar ratio = 1:1), (12) BSA–Dox (Dox concentration as in probes 10 and 11), (13) Dox; (**A**) replica on filter paper applied to bromophenol blue–stained gel (after agarose gel electrophoresis at pH 8.6); (**B**) replica on filter paper under the UV light; (**C**) the presence of Dox in the complex (BSA–CR–Dox) was confirmed chromatographically. The separation of CR–Dox mixtures was performed by Whatman 3 paper chromatography in butanol: acetic acid: water (5:1:4) solvent. Dox is seen as bright–orange fluorescence. For semiquantitative evaluation, Dox was eluted, and the fluorescence was measured (emission signal at 550 nm upon excitation with a 470 nm laser) (arrow No. “I” shows the direction of electrophoresis, and arrow “II” the direction of chromatography)—an upper fraction of Dox released during chromatography forms its complex with CR, while a lower fraction of Dox released during chromatography forms the CR–Dox–BSA complex.

It was shown that under the experimental conditions, the binding of Dox to BSA does not occur or is so weak that Dox is easily detached from the albumin (samples marked in Figure 2 as No. 6, 9, and 12 with visible Dox traveling toward the cathode). On the other hand, BSA–CR–Dox triple complexes are formed and migrate to the anode. Samples 4, 7, and 10 contain CR–Dox complexes added to albumin in 5:1, 2:1, and 1:1 molar ratios, respectively. Additionally, visible are the CR–Dox double complexes traveling faster to the anode than the triple complexes. Samples 5, 8, and 11 are CR–Dox complexes where the amount of CR is constant, and the amount of the added Dox increases. Chromatographic analysis and fluorimetric measurements showed the presence of Dox both in the complex with Congo red (CR–Dox double complex) and in the complex with BSA–CR (BSA–CR–Dox triple complex). In the Dox complexes, the differences between the proportions in the migration of the final triple complex are no longer visible, as was seen between the double complexes. BSA–CR–Dox (5:1, 2:1, and 1:1) travels just as fast as BSA–CR (Figure 2). 

**Figure 3:**
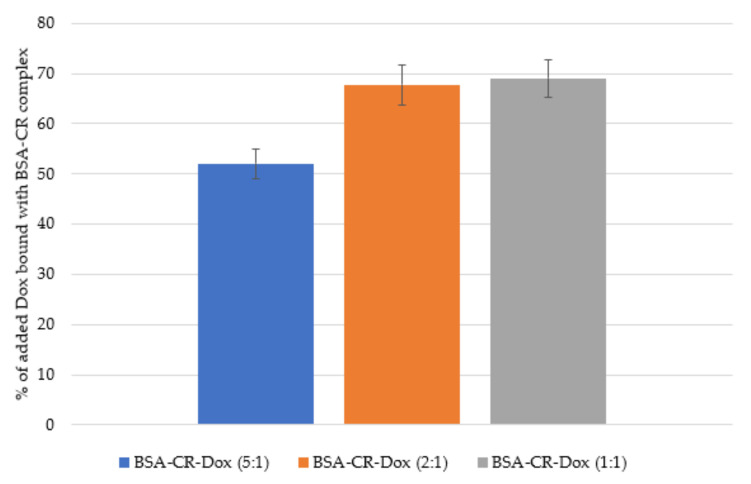
Percentage of added Dox bound with BSA–CR complex (mean ± SD, n = 3): for 5:1 molar ratio 52% ± 3%; for 2:1 molar ratio 67.8% ± 4.1%; for 1:1 molar ratio 69% ± 3.8%.

After chromatography of the individual samples and elution of the drug, the fluorimetric assessment of the amount of Dox bound in the individual complexes in relation to the amount of the drug initially administered was performed. For a CR–Dox ratio of 2:1, the same percent amount of the drug is bound to the triple complex as for a CR–Dox ratio of 1:1 (the amount of BSA and CR was the same in all samples. Only the amount of Dox added changed) (Figure 3).

### 2.2. Increasing the Amount of Dox in the BSA–CR–Dox Complex Increases the Size of This Complex. Gel–Filtration Chromatography (BioGel P–300): Elution Volumes of CR and CR–Dox Complexes with Albumin

The analysis of the size and stability of the complexes shows that the individual components of the studied complexes (BSA, CR, Dox) are eluted from the column at the same time, which was taken as proof of the complex formation. The analysis also showed that the elution volumes (V_e_) of the CR: Dox (2:1) and BSA–CR–Dox (2:1) complexes were the same (V_e_ = 0.3 mL). These systems passed through the column at the fastest rate, which means they are of comparable size. It is a surprising effect, as it means that CR forms with Dox in this proportion a complex with a size comparable to that of the protein complexed with CR–Dox (2:1). This result was confirmed using DLS analysis, which showed that both complexes (with a molar ratio of CR–Dox = 2:1), both with and without BSA, had the same size = 6.5 nm (Figure 3). The BSA–CR complex migrates faster (V_e_ = 0.6 mL) than free BSA (V_e_ = 0.7 mL), but without the Dox attached in a ratio of 2:1, it is much slower than BSA–CR–Dox (2:1). When Dox is bound to CR in a smaller amount (CR: Dox = 5:1), the complex is no longer as large and travels much more slowly through the column (V_e_ = 0.8 mL) than the complex CR: Dox = 2:1. It increases in size only after binding to BSA, but it is still smaller than the BSA–CR–Dox 2:1 complex, as it is eluted at a slower rate (V_e_ = 0.6 mL). Free CR binds to the column and does not travel through it at all (Table 1).

The influence of pH on the passage through Biogel (P–300) of individual components of the BSA–CR–Dox triple complex (molar ratio CR–Dox = 2:1) was also compared. The samples were dialyzed for 24 h at pH 7.4 and pH 5.5, respectively. They were then passed through the column, washing with buffers of appropriate pH values (7.4 and 5.5). The elution volume (V_e_) was read for each of the ternary complex components. The results are shown in Table 2. BSA, CR, and Dox of a given sample are eluted at one time, but for the 2:1 complex, a faster outflow of the triple complex can be observed at pH 5.5 than at pH 7.4, which may indicate aggregation of the systems at a reduced pH.

### 2.3. DLS Analysis

#### 2.3.1. CR–Dox (2:1) Complexes Are of the Same Size as BSA–CR–Dox Complexes (CR–Dox = 2:1)

**Figure 4:**
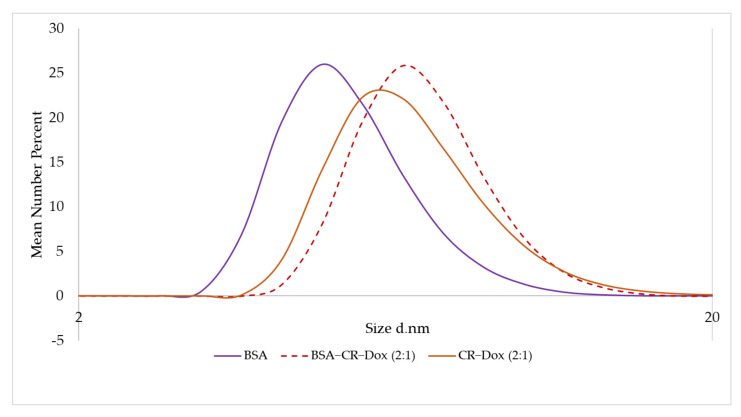
DLS analysis. Mean number (%). Hydrodynamic diameter distribution measurements: BSA 4.85 nm; CR–Dox (2:1) 6.5 nm; BSA–CR–Dox (2:1) 6.5 nm.

Using DLS analysis, it was shown that the complexes CR–Dox (molar ratio 2:1) and BSA–CR–Dox (molar ratio CR–Dox 2:1) had the same dimensions = 6.5 nm, larger than free monomeric BSA (4.85 nm) (Figure 4). These data confirmed the results obtained from gel–filtration chromatography, where both analyzed complexes had the same elution volume (V_e_) (Table 1).

#### 2.3.2. Assessment of CR–Dox Binding to Albumin—Additional Albumin Is Not Bridged with CR–Dox

**Figure 5:**
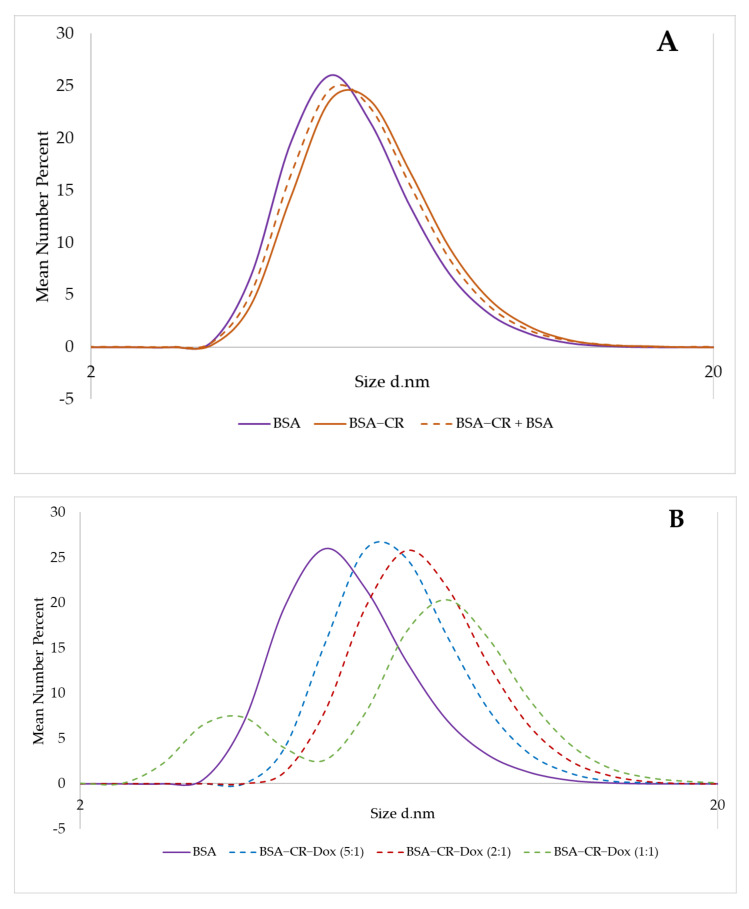

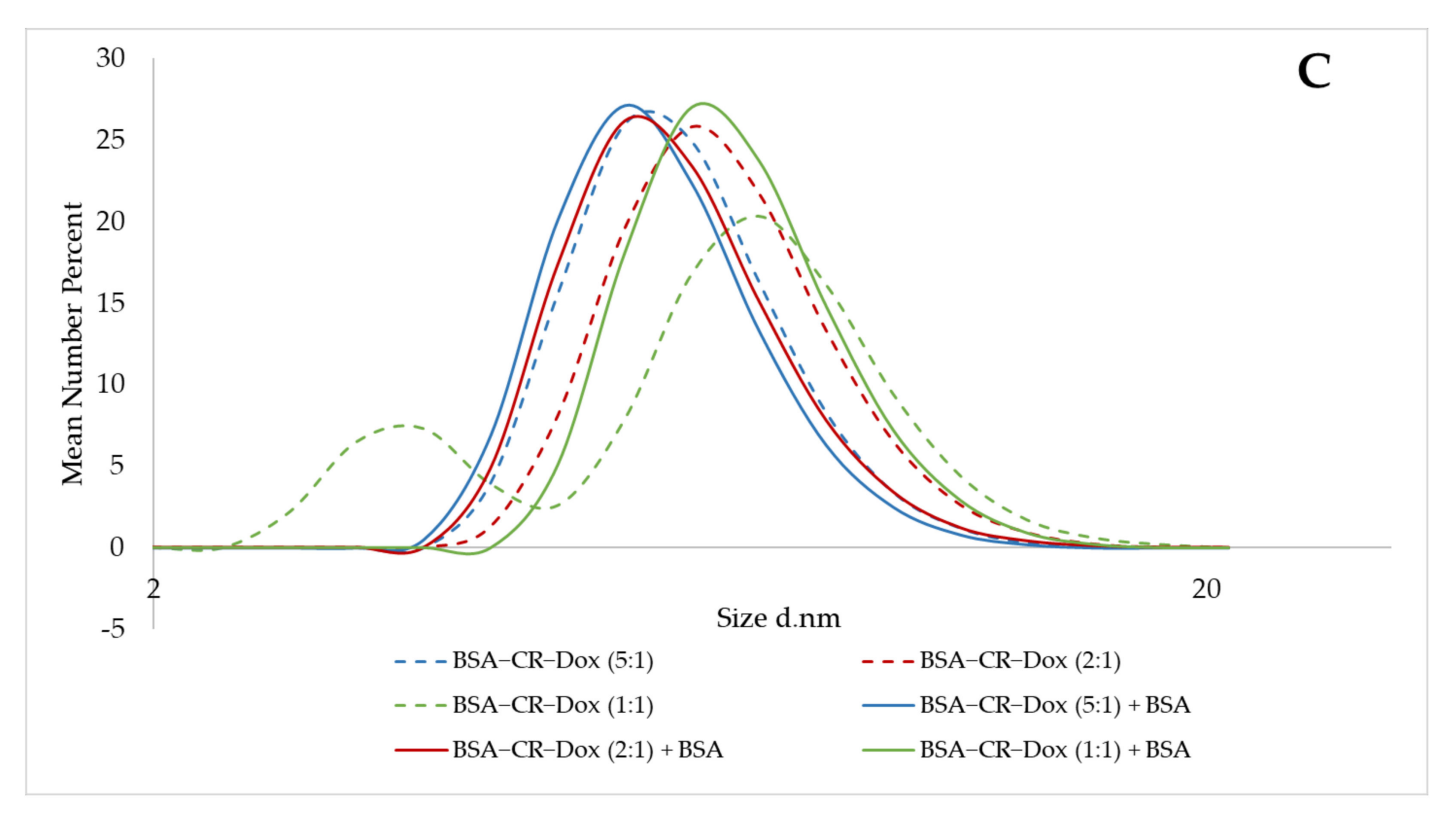
DLS analysis. Hydrodynamic diameter distribution measurements. Mean number (%). (**A**) small changes in the diameter of BSA after binding of CR (from 4.85 nm to 5.6 nm) and no significant changes after addition of an extra portion of BSA (5.6 nm before and after addition of extra BSA); (**B**) increase in the size of BSA–CR–Dox complexes after addition of an increasing amount of Dox (molar ratio of CR–Dox: 5:1, 2:1 and 1:1), respectively: 4.85 nm for free BSA, 6.5 nm for BSA–CR–Dox (5:1), 6.5 nm for BSA–CR–Dox (2:1), and 7.5 nm for BSA–CR–Dox (1:1) (additional maximum at 3.6 nm probably from an excess of unbound CR–Dox); (**C**) decrease in the size of BSA–CR–Dox complexes after addition of extra BSA, respectively: from 6.5 nm to 5.6 nm for BSA–CR–Dox (5:1), and for BSA–CR–Dox (2:1), from 7.5 nm to 6.5 nm for BSA–CR–Dox (1:1) (and disappearance of additional maximum at 3.6 nm—probably bound to extra BSA).

Increasing the amount of Dox bound to CR automatically increases the size of the CR–Dox complex. The DLS method estimated whether two BSA molecules were bridged by the bound CR–Dox complex, or whether only as the amount of Dox bound increased, the BSA–CR–Dox diameter increased due to the CR–Dox complex protruding beyond the BSA gap. The sizes of hydrodynamic diameters of the formed complexes were estimated (Figure 5).

The addition of free CR to albumin slightly increased the hydrodynamic diameter of the BSA molecule (by about 0.7 nm). The addition of excess BSA did not lead to the bridging of two BSA molecules with CR because we did not observe an increase in the hydrodynamic diameter of the molecules (Figure 5A). As the amount of doxorubicin added to CR and the binding of the CR–Dox complex thus formed with BSA increased, we observed a gradual increase in the hydrodynamic diameter of the system (Figure 5B). Adding an additional portion of BSA to the complexes thus formed did not increase the hydrodynamic diameter of the systems, and even their reduction was observed (Figure 5C). It may indicate the detachment of CR–Dox complexes protruding beyond the BSA molecule by excessively added albumin.

### 2.4. Dox Is Most Efficiently Released from the BSA–CR–Dox Complex (CR–Dox = 2:1) at pH = 5.5. Dialysis

**Figure 6:**
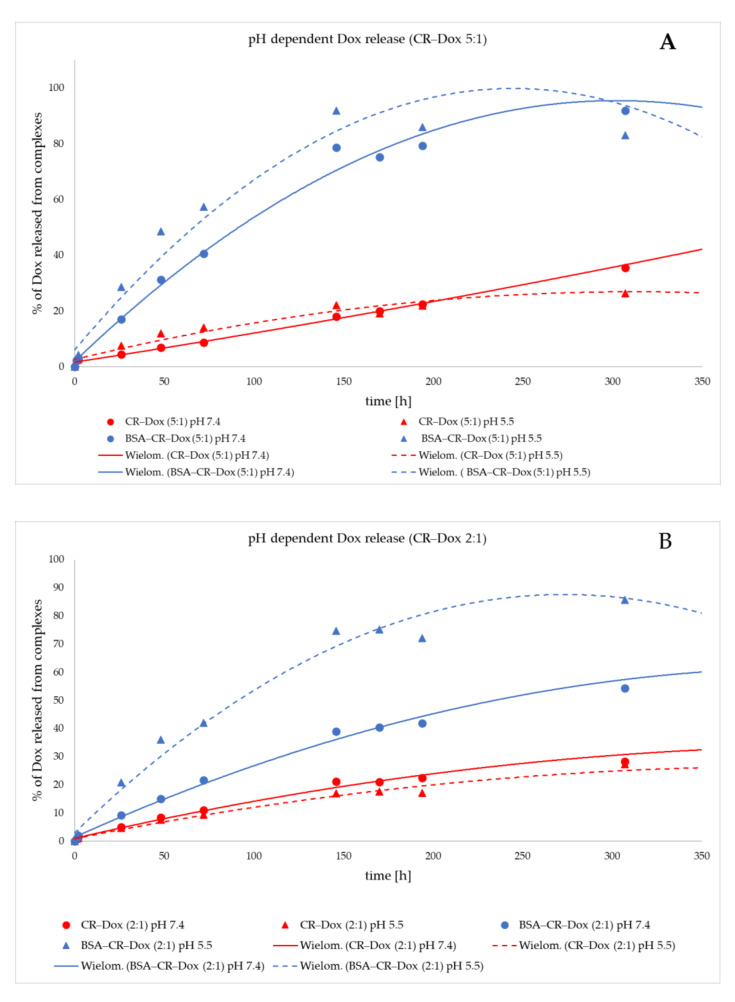

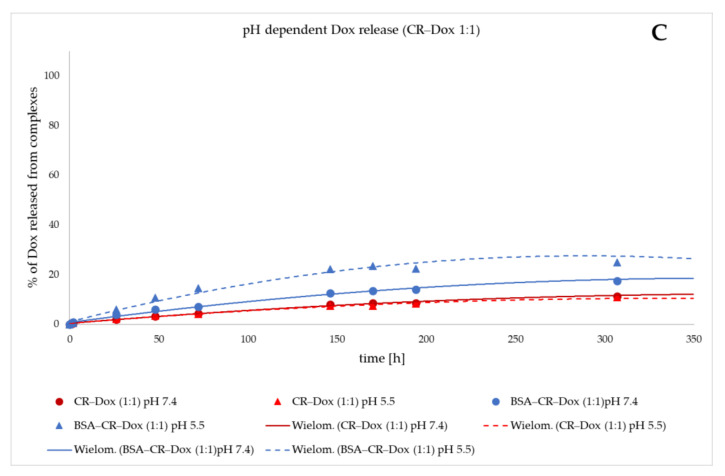
Dialysis analysis. Comparison of Dox release from CR–Dox and BSA–CR–Dox complexes at pH 7.4 and pH 5.5. Dox release rates were compared for three different molar ratios: (**A**) CR–Dox = 5:1 with or without BSA; (**B**) CR–Dox = 2:1 with or without BSA; (**C**) CR–Dox = 1:1 with or without BSA. The results were obtained in three replications.

**Figure 7:**
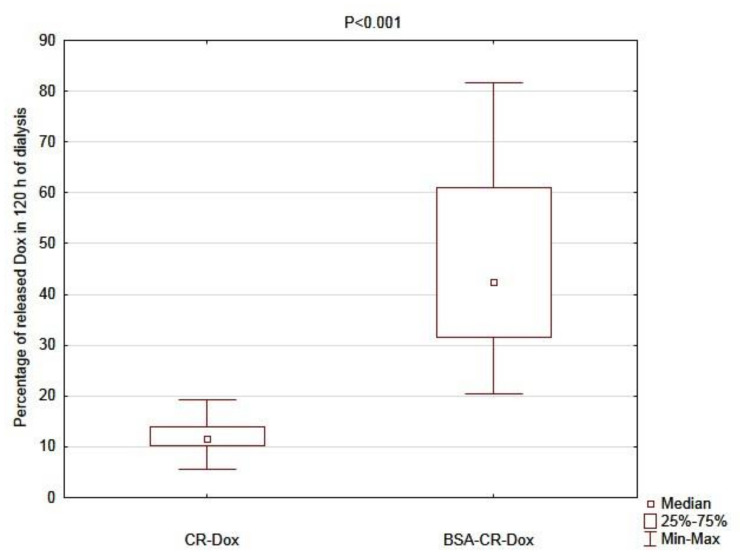
The comparison of the percentage of released Dox at 120 h of three averaged dialysis experiments between double (CR–Dox, n = 18, median = 11.52 [IQR: 10.28–13.9]) and triple (BSA–CR–Dox, n = 18, median = 42.42 [IQR: 31.65–60.91]; n—number of probes, IQR—interquartile range) complexes.

The release of Dox complexed with CR only (CR–Dox) at three different molar ratios (CR–Dox 5:1, 2:1, and 1:1) was compared. The observation period was 350 h. At all analyzed CR–Dox molar ratios, no differences in Dox release between pH 7.4 and pH 5.5 were observed. There was virtually no release of Dox from the CR–Dox complex (1:1) (red lines in the graphs; the solid line indicates Dox release at pH 7.4, and the dashed line indicates Dox release at pH 5.5 (Figure 6A–C and Figure 7A).

Additionally, at the same time, the release of Dox complexed with CR at three different molar ratios (CR–Dox 5:1, 2:1, and 1:1) and additionally bound to BSA (BSA–CR–Dox) was observed. Albumin increased the release of Dox from the complex gradually over time, reaching the value of about 70–80% during 150 h both at pH 7.4 and 5.5 if the CR–Dox molar ratio was 5:1 (Figure 6A). The best results were recorded for the 2:1 CR–Dox molar ratio in the BSA–CR–Dox complex, as the value of 70% Dox release during 150 h was achieved only at pH 5.5, while at the same time, at pH 7.4, the drug release remained at 30%. Such a system has a chance to remain stable at the physiological pH and only in the environment of the lowered pH of the neoplastic tissue gradually releases the drug (Figure 6B).

The BSA–CR–Dox complex, where the molar CR–Dox ratio was 1:1, turned out to be the most stable. Even at lowered pH, maximum drug release was maintained at 20% (Figure 6C).

#### Statistical Analysis

The analysis of mean percentage values of the released Dox from complexes in three independent dialysis experiments for each variant did not show any significant differences between them. Further, it was confirmed that the results of the three dialyses were consistent, and in the next steps, they were analyzed jointly. 

In the next step, the analysis between complexes was performed. The percentage of the released doxorubicin was significantly higher for BSA–CR–Dox than for CR–Dox when the results of each variant of dialysis were compared without taking into account differences in molar ratios and solution pH (*p* < 0.001). The graphical representation of this comparison at 120 h of dialysis is shown in Figure 7. 

**Figure 8:**
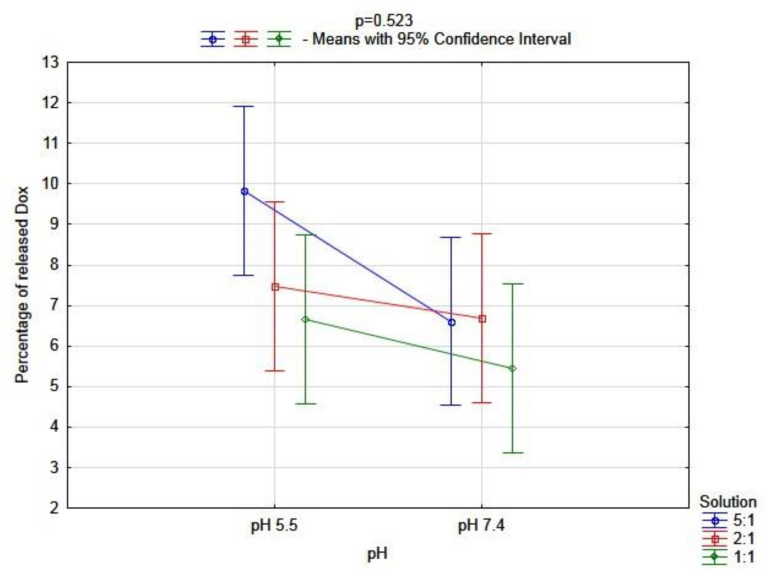
Multivariate analysis of the interaction between pH and molar ratio of CR–Dox on the percentage of the released Dox from the complex (n = 3 for each of S, in pH 5.5: S 5:1, Mean = 9.83 [95%CI: 7.75–11.91], S 2:1, Mean = 7.47 [95%CI: 5.39–9.56], S 1:1, Mean = 6.66 [95%CI: 4.57–8.74]; in pH 7.4: S 5:1, Mean = 6.61 [95%CI: 4.53–8.70], S 2:1, Mean = 6.68 [95%CI: 4–8.77], S 1:1, Mean = 5.45 [95%CI: 3.37–7.53]; n—number of probe; S—solution; 95%CI—95% Confidence Interval).

To explain the above–described phenomenon, a multivariate analysis was performed. In terms of pH (*p* = 0.079) and molar ratio (*p* = 0.191), there were no significant differences between solutions of the CR–Dox complex in the percentage of the released drug. Additionally, there was no significant interaction between those factors (*p* = 0.523) (Figure 8). It may be assumed that due to the stable character of the CR–Dox complex, the molar ratio and pH of the solution had a weaker influence on the amount of released doxorubicin.

**Figure 9:**
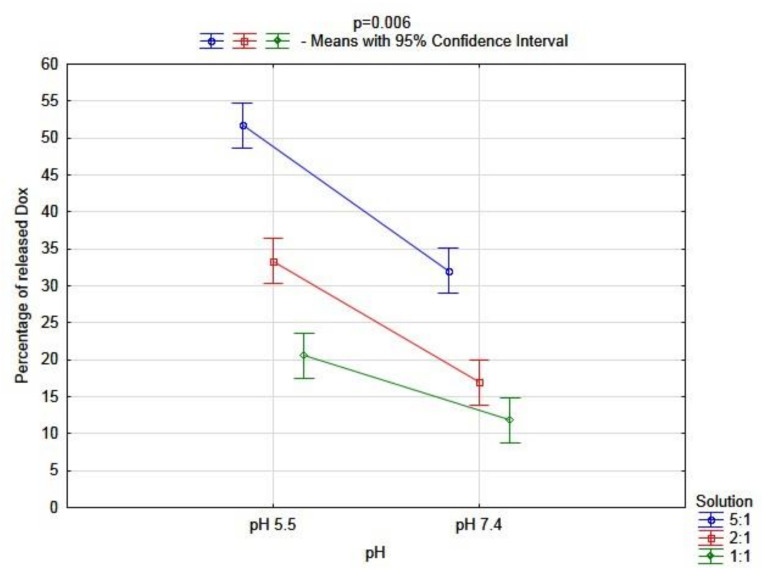
Multivariate analysis of the interaction between pH and molar ratio of BSA–CR–Dox on the percentage of the released Dox from the complex (n = 3 for each of S, in pH 5.5: S 5:1, Mean = 51.77 [95%CI: 48.72–54.82], S 2:1, Mean = 33.33 [95%CI: 30.29–36.38], S 1:1, Mean = 20.58 [95%CI: 17.54–23.63]; in pH 7.4: S 5:1, Mean = 32.06 [95%CI: 29.02–35.11], S 2:1, Mean = 16.94 [95%CI: 13.89–19.98], S 1:1, Mean = 11.84 [95%CI: 8.80–14.89]; n—number of probes; S—solution; 95%CI—95% Confidence Interval).

**Figure 10:**
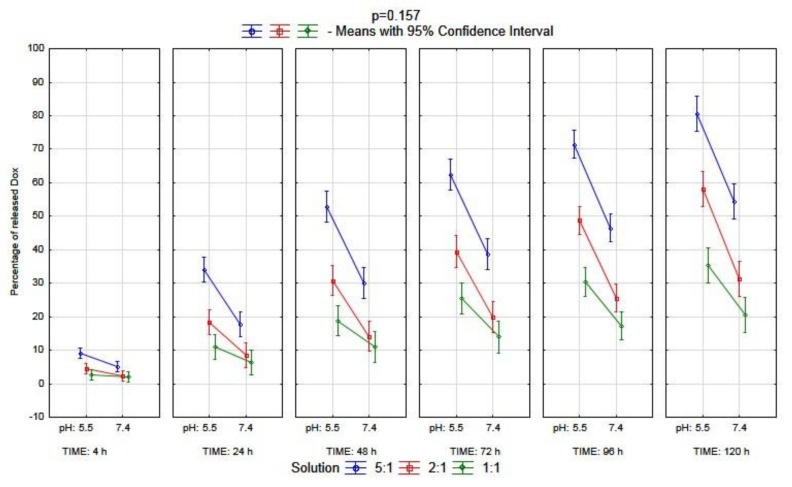
Multivariate analysis of the interaction between time, pH, and the molar ratio of BSA–CR–Dox on the percentage of the released Dox from the complex (n = 108, n = number for all of the probes).

On the other hand, the results of the analysis of the triple complex (BSA–CR–Dox) were different. The percentage of the released doxorubicin was significantly higher at pH 5.5 than at pH 7.4 (*p* < 0.001). Moreover, there were also significant differences between molar ratios (*p* < 0.001). The lowest percentage of the released drug was observed in the solution with a molar ratio of 1:1. However, those factors showed a significant interaction (*p* = 0.006, Figure 9). Due to this fact, the post hoc analysis was performed, which showed no significant differences only for comparisons: 2:1 solution pH 5.5 vs. 5:1 solution pH 7.4 (*p* = 0.985); 2:1 solution pH 7.4 vs. 1:1 solution pH 5.5 (*p* = 0.477); 2:1 solution pH 7.4 vs. 1:1 solution pH 7.4 (*p* = 0.177). For the rest of the comparisons, the differences were significant. The whole multivariate analysis for the triple complex, including all three factors (molar ratio, pH, and each moment during dialysis), is presented in Figure 10.

### 2.5. Changes in the UV/Vis Spectrum as a Result of Lowering the pH Indicate the Decomposition of the Complex (Three Different Molar Ratios)

**Figure 11:**
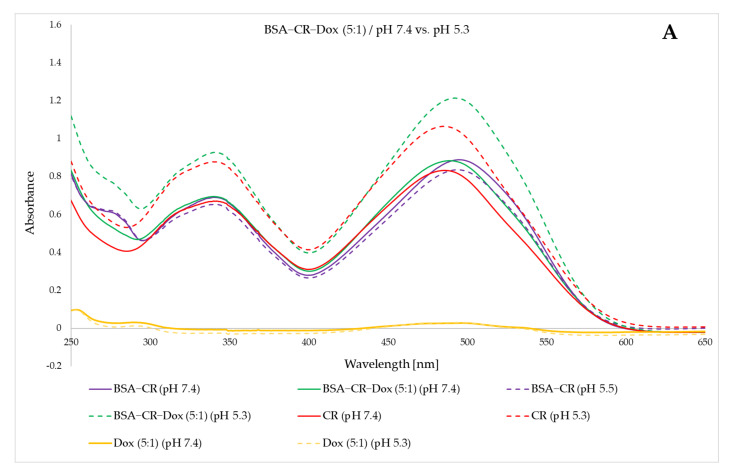

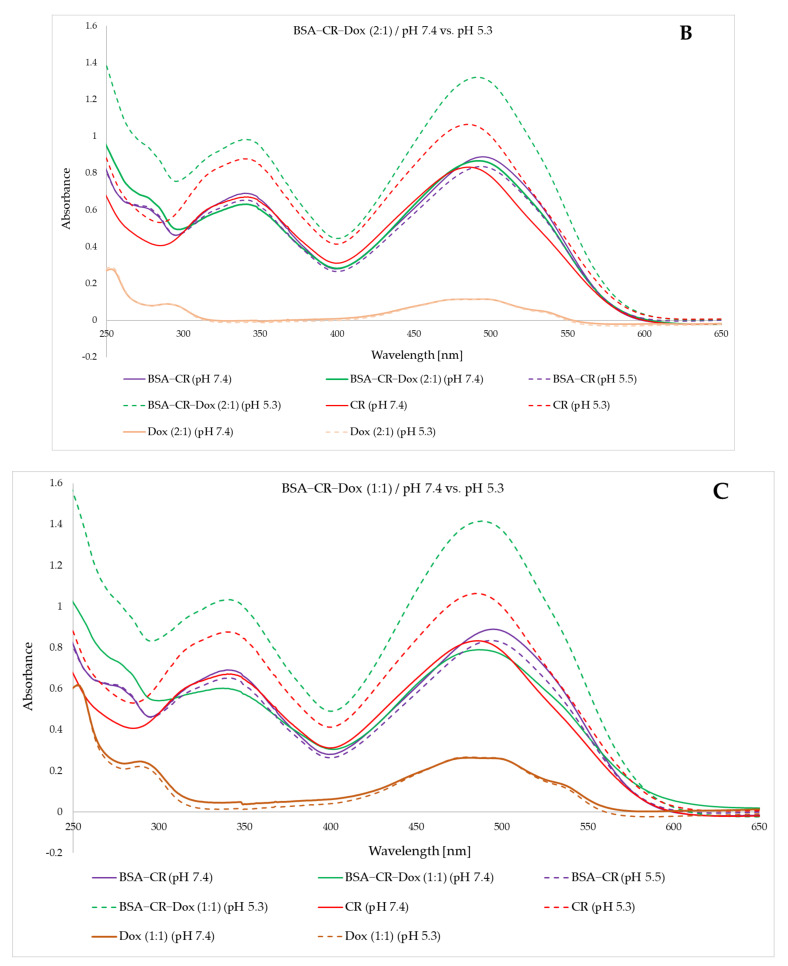
The effect of the breakdown of BSA–CR–Dox complexes (analyzed 3 molar proportions of CR–Dox) accompanying the changes of pH from 7.4 to 5.3. The spectra of the complexes at pH 5.3 are marked with dashed lines. The spectra of the complexes at pH 7.4 are marked with solid lines. (**A**) spectra of the BSA–CR–Dox complex (for a 5:1 molar ratio of CR–Dox); (**B**) spectra of the BSA–CR–Dox complex (for a 2:1 CR–Dox molar ratio); (**C**) spectra of the BSA–CR–Dox complex (for the molar ratio of CR–Dox 1:1).

A composite graph shows the BSA–CR–Dox spectra at three molar ratios of CR–Dox (5:1, 2:1, and 1:1) at two different pH values (7.4 and 5.3). The formation of BSA–CR–Dox complexes was observed at pH 7.4, as all three complexes had a spectrum lower than or at the same level as BSA–CR (Figure 11). Lowering the pH in all cases increased absorbance, which was interpreted as the release of Dox and CR from albumin. Figure 11A–C show successive changes in the spectra depending on the pH separately for the analyzed three BSA–related CR–Dox ratios.

### 2.6. Change in Size of the BSA–CR–Dox System during a Smooth pH Change (pH Range 7.4 to 4.2). DLS

**Figure 12:**
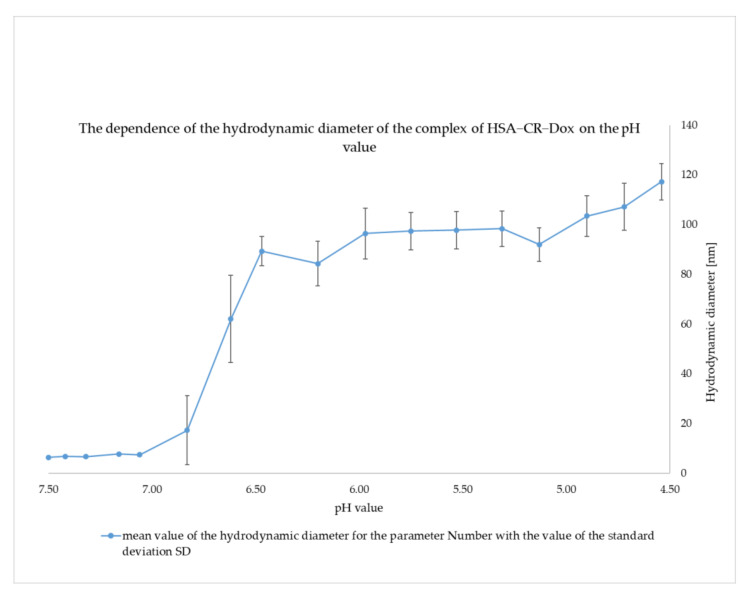
The dependence of the hydrodynamic diameter of the complex of has–CR–Dox on the pH value (mean ± SD, n = 10).

The DLS method was used to assess the size of hydrodynamic diameters, evaluate the tendency to aggregate, and compare the stability of the studied complexes. The triple complex BSA–CR–Dox (molar ratio CR–Dox = 5:1, molar ratio CR:BSA = 10:1) was investigated by analyzing the continuous change of pH from 7.4 to 4.5. The initial size of albumin with complexed CR and Dox was 6.5 nm. During the gradual lowering of the pH, it was observed that in the pH range 6.8–6.5, there was a visible, distinct step change indicative of aggregation (BSA–CR–Dox aggregates when sizes between 85 nm and 120 nm appear). This state was maintained, as the pH was further reduced to 4.5 (Figure 12). This result is consistent with the above–presented observation of differences in the size of the complexes after lowering the pH from 7.4 to 5.5, as determined by the gel–filtration chromatography method. A possible explanation for this result is that the strip of Congo red protruding beyond the albumin begins to aggregate with other CR molecules and stick the protein molecules together. This probably facilitates the release of Dox, which starts showing up in the samples.

## 3. Discussion

The binding of various drugs to albumin is described in the literature. By chemical cross–linking, it is possible to obtain micro– or nanoparticles from albumin solutions, which are used in medicine. Enclosing pharmacologically active substances in specially prepared spheres allows, first of all, for targeted therapy, but it also minimizes side effects and increases the solubility of hydrophobic drugs [32]. Most often, the production of albumin–based nanoparticles is based on the following methods: desolvation, emulsification, thermal gelling, spray drying, and the most common technology, which is used to bind hydrophobic drugs. It consists of mixing a hydrophobic drug suspended in an oil phase with an aqueous albumin solution and homogenization by passing the resulting product through a narrow nozzle. As a result of this technology, nanoparticles with a diameter of about 130 nm are obtained [33]. Albumin drug delivery systems are currently being developed for highly hydrophobic therapeutic substances to improve their solubility by eliminating toxic solvents. The group of albumin–bound compounds includes hydrophobic cytostatics, e.g., methotrexate (MTX–HSA); they have exhibited promising efficacy in various animal models and have undergone phase I/II clinical studies for further investigation [34,35,36]. Another cytostatic paclitaxel (nab–paclitaxel, manufactured by Abraxane^®^ (Celgene, NJ, USA)) was approved by the United States Food and Drug Administration (FDA) also in combination with indocyanine green. It is worth emphasizing that this dye is approved for tissue imaging [37,38]. A similar dye tested by our team, Evans Blue (EB), approved for blood volume measurements, is a supramolecular dye, similar in structure to CR but more polar. It is a safer version of the Congo Red model used in this work [39,40,41]. Albumin also binds: ABI–008 (nab docetaxel) [42], ABI–009 (nab–rapamycin—sirolimus) [43], ABI–011 (nab–synthetic analogue of thiocolchicine) [44], and Nab–lapatinib [45].

Additionally, albumin systems are used to bind water–soluble cytostatics (cisplatin, doxorubicin, phthalocyanine). In these systems, albumin acts as a protective capsule, at the same time providing the possibility of targeted transport to a specific place after the application of additional enriching substances on the protein surface [46,47,48]. 

Cisplatin is an interesting example of a drug that was developed based on platinum derivatives with different aliphatic tail lengths. The inspiration was to imitate the amphiphilic structure of fatty acids, non–covalently interacting with albumin. This is an example of the use of non–covalent interactions in a protein–small–molecule interaction [46].

The anti–cancer agent, doxorubicin (Dox), although effective in the treatment of numerous cancers, is toxic to the heart, brain, liver, and kidney [49,50]. Several carriers for Dox are used: micelles, liposomes [11,51], nanotubes [52], and gold, magnetic, or silica nanoparticles [53]. There are also studies on the use of albumin as a carrier for Dox in the form of nanoparticles (human serum albumin: HSA + Dox NPs) or Dox–loaded HSA–coated iron oxide nanoparticles [54], which are even more cytotoxic than free Dox. HSA has been modified with tumor necrosis factor (TNF)–related apoptosis–inducing ligand (TRAIL) and transferrin (Tf) on the surface before binding to Dox, generating ~220 nm nanoparticles [55]. Dox is also a component of more complex therapeutic systems, such as iron oxide nanostructures enriched with HSA and Dox particles (D–HINPs, Dox–loaded HSA–coated iron oxide nanoparticles [54]). However, albumin does not bind Dox easily and directly. The preparation procedure uses ethanol, long–term mixing, or shaking [56,57]. Dox covalently binds to albumin, which in turn limits its easy release [57,58].

Our earlier experimental studies showed the binding of Congo red to Dox. Additionally, theoretical analyses showed that CR–Dox complexes are bound by BSA. Albumin is negatively charged, but inside the large cleft, it has short basic helices responsible for binding the negatively charged CR and the CR–Dox complex [28]. The results obtained in this study confirm the binding of the model drug (Dox) to CR and the formation of a large complex binding to albumin. The results showed that the CR–Dox complex was of comparable size to the BSA–CR–Dox triple complex formed. It has been shown that the more Dox is added to the BSA–bound CR, the greater the triple complex (BSA–CR–Dox) is formed (as demonstrated by the results of measurements of hydrodynamic diameters by the DLS method). This result can be interpreted as such that the CR–Dox complex binding in the BSA cleft in the case of a large amount of Dox bound begins to protrude beyond the albumin cleft, increasing its hydrodynamic diameter. However, adding an excess of extra BSA does not cause Dox to bridge the two BSA molecules. This gives hope that the administration of a CR–linked drug with BSA into the body will not result in the formation of additional aggregates with blood albumin at physiological pH.

As it is known that albumin is responsible for maintaining colloidal osmotic pressure by binding ligands [59], it is being investigated whether such a function can prevent the release of therapeutic agents. Moreover, while high albumin content naturally occurs in the blood, the released charge could be bound back to the local albumin before traveling to specific tissues. These biological mechanisms are expected to be highlighted and investigated to improve the design and formulation of albumin–based delivery systems. By increasing the capacity, affinity, and specificity between drugs and a certain subdomain of albumin, which is generally occupied by natural ligands, such as fatty acids, for example, the drug transport efficiency can be increased. Therefore, in this study, the stability and release of Dox from complexes with different molar ratios of bound components (CR–Dox vs. BSA–CR–Dox) were analyzed. It should be emphasized that the complexes presented in this work are created easily, only by mixing the ingredients. Their formation is confirmed by the results of electrophoresis, spectral analysis, gel filtration as well as chromatography, and fluorimetric analysis after elution. Dox is also easily released from them under the influence of pH changes, as indicated by the results of the dialyses performed, changes in the spectrum, and changes in the size of the complexes in DLS. The dialysis showed no difference in Dox release from CR–Dox complexes without albumin. They also showed a different degree of Dox release from the complexes depending on the starting molar ratio of albumin–bound Congo Red and Dox. Practically 80% of the bound Dox is released from complexes with a molar ratio of CR–Dox = 5:1 after 150 h in a low pH environment. However, at the same time, a slightly smaller amount of Dox is released from this complex at pH = 7.4 (60%). The higher the molar CR–Dox ratio (2:1), the higher the initial binding of Dox, but the resulting complex is so strong that the release of the drug is lower than for the CR–Dox complex (5:1); however, there is a visible difference between release at pH 5.5 and pH 7.4 (70% and 30%, respectively). For a molar ratio of CR–Dox = 1:1, the release of Dox at pH 5.5 remained at a low level of 20% (not much different from the release at pH 7.4 at the level of about 15%). These dependencies show that it is possible to regulate the binding strength of the drug and the possibility of its release. This dependence makes it possible to control the duration of the drug release, which may be lengthened or shortened, depending on the needs.

The experiment using the gel filtration method to study the stability of the complex at reduced pH showed that BSA, CR, and Dox are eluted from a given sample at one time, but in the case of the 2:1 complex, a faster outflow of the triple complex can be observed at pH 5.5 than at pH 7.4, which may indicate an aggregation of systems at a reduced pH. This experiment additionally demonstrated the protective role of albumin for CR. CR is a model compound that, in the future, could be replaced by other such Dox tape binders, which at the same time are more biocompatible than CR itself (such as the aforementioned Evans Blue). The experiment showed no non–specific, random release of CR, so it can be predicted that the carrier thus formed will be removed from the site of action upon release of the carried cargo (drug).

Probably, when lowering the pH, CR molecules bind more tightly with each other (lowering the charge). Dialysis results showed the removal of Dox from the triple complex. This observation is comparable to the result obtained during the modeling of the SWNT–CR–Dox system [60]. From the point of view of using albumin as a carrier, we can assume that a small protein, which is albumin, binds the CR–Dox complex, transports it in the body, while protecting against non–specific Dox release and CR binding, e.g., to the vascular bed. It is worth emphasizing that only the free fraction of the drug is pharmacologically active, while the bound form of the drug is usually inactive. Unbound (free) drugs can easily diffuse nonspecifically into cells. On the other hand, the drug–protein complex (in the present case, drug–CR–albumin) is large, which limits its ability to leave the vascular space and enter the cells unspecifically. Moreover, any drug–protein complex is usually too large to be filtered by the glomeruli. The only unbound drug can be filtered and excreted by the kidneys. Thus, plasma protein binding also affects the drug clearance through the kidneys, which will increase the presence of the drug in the body and thus the duration of its action [61]. Vessels surrounding the tumor are characterized by increased permeability and, in addition, BSA receptors are present on the tumor tissue. This contributes to the increased uptake of nanoparticles by the neoplastic tissue. Both the passive and active transport of albumin with related drugs can take place. We assume that after penetrating the tumor tissue, under the influence of a lowered pH, BSA–CR could aggregate, and Dox is released. It is possible that due to aggregation and size–up, the system will remain in the tumor environment for longer, gradually releasing the drug (Figure 1). 

The diameter of the gaps between the endothelial cells of the tumor capillaries is 100–1000 nm, while the diameter of the physiological gaps in the normal endothelium is only about 2–6 nm. Albumin tends to self–oligomerize and form stable dimers [62]. There is a high probability that albumin with an increased diameter after binding to CR–Dox and additionally after possible dimerization will not penetrate through healthy vessels, but it will easily penetrate the vessels surrounding the tumor. At the same time, the size of the obtained BSA–CR–Dox complexes will be close to the physiological size of the protein, in contrast to the much larger structures obtained with the Nab technology (about 130 nm).

## 4. Materials and Methods

### 4.1. Materials

Congo Red (CR, 96% purity, Aldrich Chemical Company, Inc., Milwaukee, WI 53233 USA), doxorubicin hydrochloride (Dox, 98% purity, SIGMA–ALDRICH, Co., 3050 Spruce Street, St. Louis, MO 63103, USA), albumin from bovine serum (BSA, 96% purity, SIGMA–ALDRICH, Co., 3050 Spruce Street, St. Louis, MO 63103 USA), albumin from human serum (HSA, 96% purity, SIGMA–ALDRICH, Co., 3050 Spruce Street, St. Louis, MO 63103 USA). All other reagents used were of analytical grade and were purchased from commercial sources.

### 4.2. Methods

#### 4.2.1. Preparation of BSA–CR, CR–Dox, and BSA–CR–Dox Complexes 

Initial solutions with the following concentrations were prepared: CR = 2 mg/mL (2.86 mM), BSA = 18.95 mg/mL (0.28 mM), Dox = 1.66 mg/mL (2.86 mM). The samples were prepared in 0.05 M Tris–HCl buffer/0.154 M NaCl/pH = 7.4. The CR solution was boiled at 100 °C for 2 min and then slowly cooled to room temperature for 10 min. CR prepared in this way was combined with Dox and/or with BSA in an appropriate molar ratio, and in this way, BSA–CR–Dox complexes were obtained. In each sample where BSA and CR were present, a 10–fold molar excess of CR over BSA was used. Three molar ratios of CR: Dox were analyzed = 5:1, 2:1; 1:1. The complexes were formed by mixing the individual components in the appropriate proportions and incubating them for 15 min at room temperature. The BSA–CR–Dox complex was separated from the unbound Dox by filtration on AmiconUltra filtration tubes (MWCO 50 kDa, MERCK Millipore Ltd., Tullagreen, Carrigtwohill Co., Cork, Ireland) according to the procedure described earlier [60]. The amount of Dox bound was calculated based on measuring the fluorescence of free Dox in the filtrate (Ex = 470 nm, Em = 550 nm) and reading the result from a calibration curve.

#### 4.2.2. Characterization of CR–Dox or BSA–CR–Dox Complexes

- Assessment of differences between the complexes with different molar ratios after electrophoretic separation;

Electrophoresis was performed in 1% agarose gel (0.06 M sodium barbital buffer pH 8.6). Proteins were fixed in the gel with picrate and then stained with a bromophenol blue solution. CR was removed from the gel by reduction with sodium dithionite. The presence of Dox was revealed using a UV lamp (254 nm and 366 nm).

- Chromatographic and fluorimetric evaluation of the amount of Dox bound in the complexes;

The presence of Dox in the complex (BSA–CR–Dox) was confirmed chromatographically. After electrophoresis in one dimension, the separation of CR–Dox mixtures was performed by Whatman 3 paper chromatography in butanol: acetic acid: water (5:1:4) solvent. Dox is seen as bright–orange fluorescence. For semiquantitative evaluation, Dox was eluted, and the fluorescence was measured (emission signal at 550 nm upon excitation with a 470 nm laser). The percentage of drug–protein binding via CR was assessed spectrofluorimetrically compared to the total amount of Dox added.

- Assessment of Dox binding by BSA–CR complexes—gel filtration;

Using the gel–filtration method, the elution volumes, V_e_ (in ml), of systems of different molecular weights (CR, Dox, and the CR–Dox complex, as well as BSA, BSA–CR, BSA–CR–Dox) were compared after passing through a column filled with Biogel P–300. In samples where CR–Dox co–micelles or their complexes with proteins were present, the migration rate of individual components of the complex was analyzed separately. The presence of protein in the eluate was measured by bromophenol blue staining. CR was analyzed spectrophotometrically, and Dox was assessed by fluorimetry. A low value of the elution volume for some complexes comparable to that of protein indicates the formation of large and stable co–micelles by, i.e., CR and Dox.

- Assessment of how CR-Dox binds to albumin—changes in the size of the BSA–CR–Dox systems after adding an additional portion of BSA (DLS);

The method of CR–Dox binding to albumin was analyzed using the DLS method. The band structure of CR–Dox bound in the albumin cleft increases with the addition of CR associated with the increasing amount of Dox. It was assessed whether, after lengthening the CR–Dox tape, its fragment protruding beyond the BSA gap would bind additional BSA, thereby bridging two BSA molecules, or would only increase the diameter of the BSA by extending beyond one BSA molecule. The CR–Dox complexes (in three molar ratios CR–Dox: 1:1, 2:1, 5:1) pre–bound to BSA, followed by an extra portion of BSA added, were used.

#### 4.2.3. Assessment of the Release of Dox after Lowering the pH

- Assessment of Dox release depending on the pH—dialysis;

The kinetics of Dox release from BSA–CR–Dox complex at neutral pH = 7.4 and acidic pH = 5.5 was determined by dialysis using D–TubeTM Dialyzers Mini, MWCO 12–14 kDa (Novagen, MERCK Millipore, EDM Millipore Corp., Billerica, MA, USA). Dialysis tubes were pre–saturated with CR solution, which easily binds to the cellulose membrane. This helped to avoid the possibility of non–specific removal of CR or its Dox complexes from the protein. BSA–CR–Dox samples were dialyzed to Tris–HCl buffer (0.05 M/0.154 M NaCl/pH 7.4) or to acetate buffer (0.05 M/0.154 M NaCl/pH 5.5). The experiment was carried out in closed containers with a dialysis tube inside, at room temperature, with stirring, and in the dark. At several time points (to a maximum of 120 h), samples of dialysis fluid were collected for analysis and were replaced with the same portions of the appropriate fresh buffer. The amount of Dox released was calculated based on measuring the fluorescence of free Dox in the filtrate (Ex = 470 nm, Em = 550 nm) and reading the result from a calibration curve. The % of released Dox was calculated based on the amount of Dox added to the sample. Dialysis was repeated 3 times, and the results were averaged from these 3 measurements.

- Assessment of Dox release depending on the pH—UV/VIS spectrum;

Samples were prepared as described in Section 4.2.1. Three molar ratios of CR:Dox were analyzed, namely, 5:1, 2:1; 1:1 bound to BSA (10–fold molar excess of CR over albumin). Samples were suspended in Tris–HCl buffer (0.05 M/0.154 M NaCl/pH 7.4) or in acetate buffer (0.05 M/0.154 M NaCl/pH 5.5), respectively, and incubated for 24 h.

- Assessment of the stability of the tested complexes depending on the pH—Dynamic light scattering (DLS);

The hydrodynamic diameter was measured with the use of dynamic light scattering (DLS) detector Zetasizer Nano ZSP (Malvern, UK) with laser incident beam at λ = 633 nm and a fixed scattering angle of 173° according to the procedure described earlier [60].

Continuous measurement of the hydrodynamic radius of the BSA–CR–Dox sample (in molar ratios CR–Dox = 5:1) was conducted during pH change from 7.4 to 4.5 at the interval of 0.2 pH value. A titrator device MPT–2 was applied with three independent containers for titrating solutions (0.25 M HCl for initial acidification and 0.05 M HCl for precise pH adjustment and 0.25 M NaOH) and with a degassing unit for greater efficiency and precision of titration. The BSA–CR–Dox sample, with the initial pH = 7.4, was prepared as described in Section 4.2.1. The hydrodynamic diameter was measured based on the values of numbers using a flow–through cell.

#### 4.2.4. Statistical Analysis

The differences between the release of Dox from the CR–Dox and BSA–CR–Dox complexes during dialysis were subjected to statistical analysis. Relative (to the controls) increases in Dox release from BSA–CR–Dox (in different molar ratios of CR–Dox) determined after 24 h, 48 h, 72 h, 96 h, and 120 h of dialysis in two different pH: 5.5 and 7.4, were analyzed.

The Statistica 13.0 software (StatSoft, Statistica 13.0, Tulsa, OK, USA) was used for statistical analysis. Values of continuous data were presented as the mean and standard deviation (SD) or the median and interquartile range (IQR). Continuous variables were first checked for normal distribution by the Shapiro–Wilk test. In cases with two groups of variables with other than normal distribution, the Mann–Whitney test was used, and values were presented as the median and IQR. Otherwise, for data with normal distribution, the *T*–test was applied with the mean and standard deviation (SD). To compare more than two groups, the ANOVA analysis with proper corrections was performed. For multivariate analyses of the interaction between factors, the proper ANOVA tests and the post hoc HSD Tukey test were used. For all tests, a *p* value of less than 0.05 was considered significant.

## 5. Conclusions

To conclude, a new system was obtained consisting of a supramolecular carrier—Congo red, which binds doxorubicin through intercalation. The resulting CR–Dox system is bound to albumin. As a result, a carrier system of about 6–8 nm is obtained when using albumin monomers, correspondingly larger if dimers are used. Drug release from systems with different CR–Dox molar ratios was analyzed, and the most optimal ratio (2:1) was selected, at which Dox was gradually, and thus most effectively, released at reduced pH. The resulting carrier arrangement is obtained in a very simple manner. The system has the potential to become an alternative to the existing delivery system for anti–cancer drugs. 

## Figures and Tables

**Table 1:** Gel–filtration chromatography: elution volume (V_e_) of BSA, CR, and Dox in mixtures of BSA–CR–Dox, CR–Dox, BSA–CR (1:10), and in free BSA (Biogel P–300). The molar ratios of CR–Dox in all mixtures are 2:1 or 5:1. V_e Dox free_ = 2.1 mL (not shown).

Elution Volume (V_e_)	BSA–CR–Dox (5:1)	BSA–CR–Dox (2:1)	CR–Dox (5:1)	CR–Dox (2:1)	BSA–CR	BSA
BSA [mL]	0.6	0.3	-	-	0.6	0.7
CR [mL]	0.6	0.3	0.8	0.3	0.6	-
Dox [mL]	0.6	0.3	0.8	0.3	-	-
Complex creation	YES	YES	YES	YES	YES	-

**Table 2:** Gel–filtration chromatography: elution volume (V_e_) of BSA, CR, and Dox in mixtures of BSA–CR–Dox in two pH: 7.4 and 5.5 (Biogel P–300). The molar ratios of CR–Dox in all mixtures are 2:1.

Elution Volume (V_e_)	BSA–CR–Dox (2:1) pH 7.4	BSA–CR–Dox (2:1) pH 5.5
BSA [mL]	0.6	0.4
CR [mL]	0.6	0.4
Dox [mL]	0.6	0.4
Complex creation	YES	YES

## Data Availability

The data presented in this stude are available on request from the corresponding author.
